# Parental expectations for physical activity and adolescents’ exercise behavior: a chain mediation of basic psychological needs and exercise motivation moderated by gender

**DOI:** 10.3389/fpsyg.2026.1964799

**Published:** 2026-10-19

**Authors:** Shikai Feng

**Affiliations:** Department of Physical Education, Xinyang Normal University, Xinyang, Henan, China

**Keywords:** adolescents, basic psychological needs, chain mediation, exercise motivation, gender moderation, parental expectations, physical activity, self-determination theory

## Abstract

**Background:**

Parental expectations play a key role in shaping adolescents’ health behaviors, yet the mechanisms linking them to exercise behavior remain insufficiently understood. Grounded in Self-Determination Theory (SDT) and the Expectancy-Value Model, this study tests a chain mediation model in which parental expectations for physical activity are associated with adolescents’ exercise behavior through basic psychological need satisfaction and exercise motivation, with gender as a moderator.

**Methods:**

A cross-sectional survey was conducted among 518 Chinese adolescents (Mage = 15.32 years, S*D* = 1.74; 50.4% female) from four middle schools in Henan Province, China. Participants completed measures of perceived parental expectations for physical activity, basic psychological need satisfaction in exercise (BPNSS), the Behavioral Regulation in Exercise Questionnaire-2 (BREQ-2), and the International Physical Activity Questionnaire Short Form (IPAQ-SF). Chain mediation and gender moderation were tested with structural equation and multi-group modeling.

**Results:**

Parental expectations were associated with adolescents’ exercise behavior both directly (*β* = 0.11, *p* < 0.01) and indirectly [total indirect effect *β* = 0.13, 95% CI (0.08, 0.17)], including the sequential pathway through basic psychological need satisfaction and autonomous exercise motivation [*β* = 0.04, 95% CI (0.02, 0.05)]. The total indirect effect accounted for 52.6% of the total effect, with the chain-specific pathway accounting for 15.7%. The chain-mediated indirect effect was stronger for males [effect = 0.06, 95% CI (0.03, 0.09)] than females [effect = 0.02, 95% CI (0.01, 0.04)]; the between-group difference was 0.04, 95% bootstrap CI [0.01, 0.07]. Females reported higher perceived parental expectations (*d* = −0.26) and autonomous motivation (*d* = −0.22), yet the motivational pathway linking parental expectations to exercise behavior was stronger among males.

**Conclusion:**

Basic psychological need satisfaction and autonomous exercise motivation constitute two interlinked motivational mechanisms that statistically account for the association between parental expectations and adolescents’ exercise behavior, and this pathway was stronger among males. Because the data are cross-sectional, these findings represent associations compatible with the proposed theoretical sequence rather than demonstrated causal effects. Heightened expectations alone may be insufficient; fostering need satisfaction and autonomous motivation may be important for sustained exercise engagement, particularly among female adolescents.

## 1 Introduction

Physical inactivity among adolescents has emerged as a pressing global public health challenge. The World Health Organization (2020) recommends that adolescents aged 11–17 years accumulate at least 60 min of moderate-to-vigorous physical activity (MVPA) daily, yet approximately 81% of adolescents worldwide fail to meet this guideline. In China, the situation is particularly concerning: recent national surveillance data indicate that only 22.6% of Chinese adolescents achieve the recommended levels of physical activity, with a marked decline observed during the middle school years (Chen et al., 2020). This widespread inactivity not only elevates the risk of non-communicable diseases but also compromises adolescents’ psychological well-being, cognitive development, and academic performance (Biddle et al., 2019).

Among the myriad factors influencing adolescent physical activity, parental influence has been consistently identified as one of the most proximal and powerful determinants. A landmark meta-analysis of 115 studies by Yao and Rhodes (2015) reported a moderate overall effect of parental support on child and adolescent physical activity (*r* = 0.38), a finding that has been replicated and extended in more recent meta-analytic work (Su et al., 2022), with parental encouragement, logistical support, and co-activity each contributing uniquely to youth activity levels. Qualitative and quantitative reviews have similarly shown that parents shape adolescents’ physical activity through multiple channels: role modeling, instrumental support, emotional encouragement, and the communication of values and expectations regarding the importance of an active lifestyle (Edwardson and Gorely, 2010; Beets et al., 2010).

Among the diverse forms of parental influence, parental expectations—defined as parents’ beliefs and aspirations regarding their children’s engagement in physical activity—have received comparatively less empirical attention than overt parental support behaviors. Yet theoretical frameworks, particularly the Expectancy-Value Model of parental socialization (Fredricks and Eccles, 2004, 2005), posit those parental expectations function as a critical socialization mechanism. According to this model, parents who hold higher expectations for their children’s physical activity are more likely to communicate these expectations through encouragement, provide activity-promoting resources and opportunities, and serve as active role models. In turn, children internalize these parental messages, shaping their self-perceptions, values, and ultimately their behavioral choices regarding physical activity (Eccles and Harold, 1991).

Despite the theoretical plausibility of the parental expectation–adolescent exercise behavior link, critical mechanistic questions remain unresolved. Specifically, through what psychological processes do parental expectations become internalized and translated into adolescents’ actual exercise behavior? Drawing on Self-Determination Theory (SDT; Deci and Ryan, 1985; Ryan and Deci, 2000, 2017), we propose that two key psychological constructs—basic psychological need satisfaction and exercise motivation—function as sequential mediators in this relationship.

SDT contends that the satisfaction of three basic psychological needs—autonomy (the need for volitional engagement), competence (the need to feel effective), and relatedness (the need for social connection)—constitutes the psychological nutriments essential for autonomous motivation and optimal functioning (Ryan and Deci, 2000). When parents communicate expectations in a need-supportive manner—for example, by acknowledging adolescents’ perspectives, providing meaningful rationales, and offering choices rather than exerting controlling pressure—adolescents are more likely to experience need satisfaction in the exercise domain. This need satisfaction, in turn, fosters autonomous exercise motivation, defined as engaging in physical activity out of genuine interest, enjoyment, or personal valuation of its benefits, rather than external contingencies or internal pressures (Deci and Ryan, 2000).

A robust body of evidence in physical education and exercise settings has demonstrated the sequential SDT pathway: need-supportive environments predict basic psychological need satisfaction, which predicts autonomous motivation, which in turn predicts adaptive behavioral outcomes including physical activity participation (Standage et al., 2003; Ntoumanis, 2001; Vasconcellos et al., 2020). However, the application of this motivational sequence to the parental expectation context remains unexplored. It is plausible that the extent to which parental expectations facilitate or undermine adolescents’ exercise behavior depends critically on whether these expectations are perceived as supportive of—or thwarting to—adolescents’ basic psychological needs. Recent work using device-based measurement has further corroborated this sequential pathway in adolescent populations (Emm-Collison et al., 2024).

Furthermore, the potential moderating role of gender in the parental expectation–exercise behavior pathway has been largely overlooked. The Expectancy-Value Model posits that parents hold gender-differentiated beliefs about their children’s competence and the value of physical activity, with parents generally perceiving sports and exercise as more important for sons than for daughters (Fredricks and Eccles, 2005; Eccles and Harold, 1991). Consistent with this proposition, Yao and Rhodes’ (2015) meta-analysis found stronger father-son modeling effects (*r* = 0.29) compared to mother-son effects (*r* = 0.19), and research has documented gender disparities in parental encouragement for physical activity (Trost et al., 2003). These findings raise the possibility that the motivational mechanisms linking parental expectations to exercise behavior may operate differently for male and female adolescents.

The present study thus aims to address three key research gaps. First, we test a chain mediation model in which parental expectations for physical activity predict adolescents’ exercise behavior through the sequential mediation of basic psychological need satisfaction and autonomous exercise motivation. Second, we examine both the direct and indirect pathways through which parental expectations influence exercise behavior. Third, we investigate whether gender moderates the chain-mediated pathway. We hypothesized that: (H1) parental expectations positively predict adolescents’ exercise behavior; (H2) basic psychological need satisfaction and autonomous exercise motivation sequentially mediate the parental expectation–exercise behavior relationship; and (H3) the chain-mediated pathway is moderated by gender, such that the indirect effect is stronger for males than females.

## 2 Theoretical framework and literature review

### 2.1 Parental expectations and adolescent physical activity

The Expectancy-Value Model of parental socialization (Fredricks and Eccles, 2004) provides a comprehensive framework for understanding how parents influence their children’s motivational and behavioral development in achievement domains including sport and physical activity. The model identifies three primary parental roles: provider (facilitating access to activities through logistical and financial support), interpreter (communicating beliefs, values, and expectations about the activity), and role model (demonstrating behaviors and attitudes). Through these roles, parents’ expectations regarding their children’s ability and the value they place on physical activity are transmitted to children, shaping children’s self-perceptions of competence, subjective task value, and ultimately their participation decisions (Fredricks and Eccles, 2005).

Empirical support for the parental expectation–physical activity link, while less extensive than research on parental support behaviors, is accumulating. Trost et al. (2003) evaluated a model of parental influence in a sample of 380 adolescents and found that parental physical activity orientations—including beliefs about the importance of physical activity—predicted parental support, which in turn predicted adolescents’ physical activity both directly and indirectly through adolescents’ self-efficacy. More recently, Sallis et al. (2000) identified parental influence as one of the most consistent correlates of adolescent physical activity in their landmark review. A large-scale study by Babic et al. (2014) demonstrated that parental expectations and beliefs about children’s physical competence were significantly associated with children’s physical self-concept, which in turn predicted physical activity behavior.

In the Chinese context, parental expectations carry particular cultural weight. Chinese culture traditionally emphasizes filial piety and academic achievement, often at the expense of physical activity and leisure pursuits. However, with China’s national policies promoting youth physical fitness—including the Healthy China 2030 initiative and the Double Reduction policy—parental attitudes toward physical activity are undergoing transformation. Understanding how Chinese parents’ expectations regarding physical activity shape their adolescents’ exercise behavior is therefore of both theoretical and practical significance.

### 2.2 Basic psychological needs as a mediating mechanism

Self-Determination Theory posits that environmental factors influence motivation and behavior through their impact on basic psychological need satisfaction (Deci and Ryan, 2000; Ryan and Deci, 2017). When social contexts support autonomy, competence, and relatedness, individuals experience greater need satisfaction, which catalyzes autonomous motivation and well-being. This proposition has received consistent empirical support across diverse life domains. In physical education settings, Vasconcellos et al. (2020) reported in a comprehensive meta-analysis of 265 studies that perceived autonomy support from teachers positively predicted basic psychological need satisfaction (*β* = 0.44), which in turn predicted autonomous motivation (*β* = 0.50).

Extending this logic to the parental domain, parental expectations that are communicated in a need-supportive manner—characterized by acknowledging the adolescent’s perspective, providing meaningful choices, and offering competence-affirming feedback—should facilitate basic psychological need satisfaction. Conversely, expectations delivered in a controlling manner—involving pressure, conditional regard, or coercion—may thwart need satisfaction and undermine autonomous motivation (Grolnick and Ryan, 1989). Given that Chinese parenting practices have been characterized by both high expectations and, in some contexts, controlling parenting styles, understanding the need-satisfaction pathway is particularly relevant for this population.

### 2.3 The role of exercise motivation quality

A central tenet of SDT is the distinction between autonomous and controlled motivation. Autonomous motivation comprises intrinsic regulation (engaging in an activity for inherent enjoyment) and identified regulation (engaging because one personally values the activity’s outcomes). Controlled motivation encompasses introjected regulation (engaging to avoid guilt or maintain self-esteem) and external regulation (engaging due to external demands or rewards) (Deci and Ryan, 2000). Research has consistently demonstrated that autonomous motivation, relative to controlled motivation, predicts greater behavioral persistence, more frequent physical activity participation, and enhanced psychological well-being (Standage et al., 2003; Ntoumanis, 2001).

The Relative Autonomy Index (RAI; Mullan et al., 1997) provides a composite index of motivational quality by weighting each regulatory style along a continuum from controlled to autonomous. Higher RAI scores reflect a more autonomous motivational orientation. In the present study, we operationalize exercise motivation quality using the RAI derived from the Behavioral Regulation in Exercise Questionnaire-2 (BREQ-2; Markland and Tobin, 2004). We propose that basic psychological need satisfaction facilitates the internalization of exercise motivation, shifting the motivational orientation from controlled to autonomous, which in turn promotes sustained exercise engagement.

### 2.4 Gender as a moderator

Gender differences in physical activity participation are well-documented, with female adolescents consistently reporting lower levels of physical activity than males across diverse cultural contexts (Sallis et al., 2000; Biddle et al., 2019). Explanations for this gender gap have focused on differential socialization practices: parents are more likely to encourage physical activity in sons, perceive sports as more important for boys, and provide greater activity-related resources to male children (Fredricks and Eccles, 2005; Eccles and Harold, 1991).

Beyond mean-level differences, the motivational processes linking parental influence to exercise behavior may differ qualitatively by gender. Yao and Rhodes (2015) found that parental support showed a stronger association with boys’ physical activity (*r* = 0.41) than girls’ (*r* = 0.34), although this difference did not reach statistical significance. Trost et al. (2003) reported that the parental support–youth physical activity pathway was more robust for boys. These patterns may reflect gender-role socialization: because physical activity is normatively more congruent with the male gender role, boys may be more receptive to and responsive to parental expectations regarding exercise participation. Conversely, for girls, competing social pressures and gender-role expectations may attenuate the translation of parental expectations into actual exercise behavior (Edwardson and Gorely, 2010).

### 2.5 The present study: a moderated chain mediation model

Integrating the Expectancy-Value Model of parental socialization with Self-Determination Theory, the present study proposes and tests a moderated chain mediation model. Specifically, we hypothesize that parental expectations for physical activity influence adolescents’ exercise behavior through two sequential psychological mechanisms: basic psychological need satisfaction (Mediator 1) and autonomous exercise motivation (Mediator 2). Furthermore, we examine whether gender moderates this chain-mediated pathway, expecting stronger indirect effects for male adolescents (Figure 1). By simultaneously examining both the mediating mechanisms and boundary conditions of parental expectation effects, this study advances theoretical understanding of how and for whom parental expectations translate into adolescent exercise behavior.

**Figure 1:**
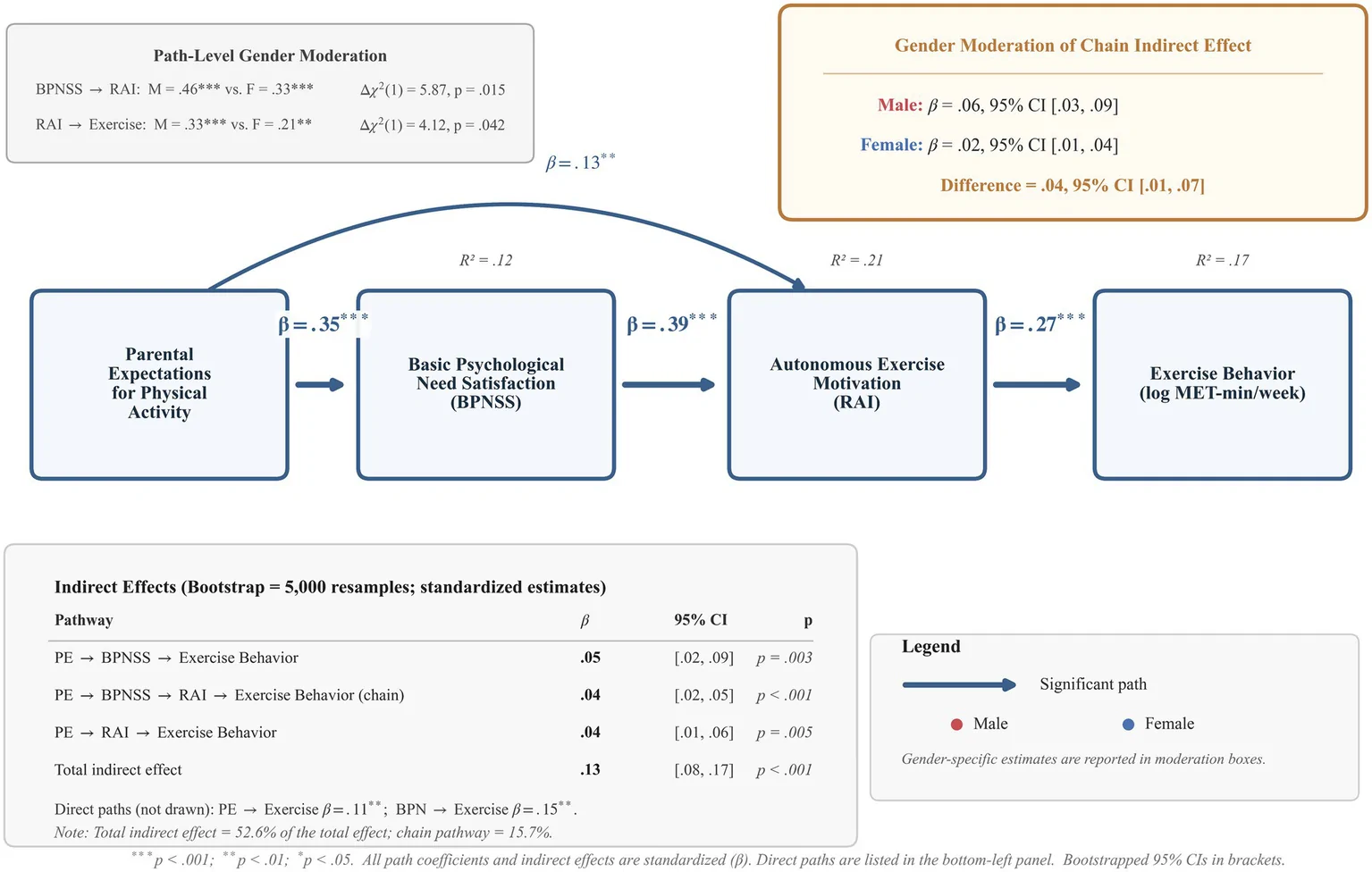
Moderated chain mediation model displaying standardized path coefficients for the full sample (*N* = 518). The two direct paths (PE → Exercise and BPN → Exercise) are reported in the bottom panel. The path moderation panel shows gender-differentiated coefficients (male values first, female second; see Table 4 for the complete set of gender-specific paths). The indirect effects table summarizes bootstrap mediation results (5,000 resamples, bias-corrected 95% CI). ***p < 0.001; **p < 0.01; *p < 0.05.

## 3 Materials and methods

### 3.1 Participants and procedure

A cross-sectional survey design was employed. Data were collected between October and December 2025 from four public middle schools in Xinyang City, Henan Province, central China. Schools were selected using stratified random sampling to ensure representation across urban and suburban districts. Following approval from the Institutional Review Board of Xinyang Normal University, written informed consent was obtained from the legal guardians of all participating adolescents, and verbal assent was obtained from the adolescents themselves.

The initial sample comprised 560 students from grades 7 through 11. After excluding participants with excessive missing data (>20% of items; *n* = 23), straight-lining response patterns (*n* = 12), and implausible IPAQ values exceeding 15,000 MET-min/week (*n* = 7), the final analytic sample consisted of 518 adolescents (Mage = 15.32 years, S*D* = 1.74, range = 12–18 years; 50.4% female; 56.8% urban, 43.2% suburban). The sample was predominantly Han Chinese (96.1%). The majority of participants (78.6%) lived in two-parent households, while 14.3% lived with one parent, and 7.1% lived with grandparents or other relatives.

Questionnaires were administered in classroom settings during regular school hours by trained research assistants. To minimize social desirability bias, participants were assured of the confidentiality and anonymity of their responses and were seated with adequate spacing. The questionnaire battery took approximately 20–25 min to complete.

### 3.2 Measures

#### 3.2.1 Perceived parental expectations for physical activity

Perceived parental expectations for physical activity were assessed using a 6-item scale adapted from the parental expectation measures developed by Fredricks and Eccles (2005) and Trost et al. (2003). Items were reworded to focus specifically on the physical activity domain and were translated into Chinese following standard back-translation procedures. Specifically, the items were translated into Chinese by two bilingual researchers specializing in sport psychology and independently back-translated into English by a third bilingual translator who was blind to the original items. Discrepancies between the original and back-translated versions were reviewed and resolved through discussion by a panel consisting of the translators and the author. A pilot administration with 32 adolescents from a non-sampled school confirmed the clarity and comprehensibility of all items, and minor wording refinements were made accordingly. Sample items include: “My parents expect me to participate in sports or exercise regularly,” “My parents believe it is important for me to be physically active,” and “My parents want me to achieve a high level of physical fitness.” Participants responded on a 5-point Likert scale ranging from 1 (strongly disagree) to 5 (strongly agree). Higher scores indicate higher perceived parental expectations. Confirmatory factor analysis supported the unidimensional structure of the scale (CFI = 0.96, RMSEA = 0.052). Internal consistency in the present sample was good (*α* = 0.86).

#### 3.2.2 Basic psychological need satisfaction in exercise

Satisfaction of basic psychological needs in the exercise context was assessed using an adapted version of the Basic Psychological Need Satisfaction Scale (BPNSS; adapted from Standage et al., 2003). The scale comprises 12 items measuring autonomy (4 items; e.g., “I feel free to exercise in my own way”), competence (4 items; e.g., “I feel confident in my ability to exercise”), and relatedness (4 items; e.g., “I feel connected to the people I exercise with”). Items were rated on a 5-point Likert scale from 1 (strongly disagree) to 5 (strongly agree). Consistent with prior SDT research employing composite need satisfaction indices (Vasconcellos et al., 2020), an overall need satisfaction score was computed by averaging across all 12 items. This choice was grounded in both theory and evidence: SDT conceptualizes the three needs as complementary psychological nutriments that operate conjointly (Ryan and Deci, 2017), and the three subscales were strongly intercorrelated in the present sample (rs = 0.52–0.61), supporting the psychometric defensibility of the composite. Internal consistency was *α* = 0.90.

#### 3.2.3 Exercise motivation

Exercise motivation was assessed using the Chinese version of the Behavioral Regulation in Exercise Questionnaire-2 (BREQ-2; Markland and Tobin, 2004). The BREQ-2 is a 19-item self-report measure assessing five types of behavioral regulation: amotivation (4 items), external regulation (4 items), introjected regulation (3 items), identified regulation (4 items), and intrinsic regulation (4 items). Participants responded on a 5-point Likert scale from 0 (not true for me) to 4 (very true for me). Following established procedures (Mullan et al., 1997), a Relative Autonomy Index (RAI) was calculated: RAI = (amotivation × − 3) + (external × − 2) + (introjected × − 1) + (identified × 2) + (intrinsic × 3). Higher RAI scores indicate more autonomous motivation. The BREQ-2 subscales demonstrated acceptable internal consistency (*α* = 0.77–0.87).

#### 3.2.4 Exercise behavior

Exercise behavior was measured using the International Physical Activity Questionnaire Short Form (IPAQ-SF; Craig et al., 2003). The IPAQ-SF assesses the frequency (days per week) and duration (minutes per day) of walking, moderate-intensity activity, and vigorous-intensity activity over the preceding seven days. Total weekly physical activity was calculated as metabolic equivalent of task (MET) minutes per week using the standard IPAQ scoring protocol. Following IPAQ data processing guidelines, MET-min/week values were log-transformed to address positive skewness. The IPAQ-SF has demonstrated acceptable test–retest reliability (pooled *ρ* = 0.76) and criterion validity against accelerometry (*ρ* = 0.30) across diverse populations (Craig et al., 2003).

#### 3.2.5 Demographics

Participants reported their age, gender, grade level, residential area (urban vs. suburban), and family composition (two-parent, single-parent, or other household type).

### 3.3 Data analysis

Data were screened for normality, outliers, and missing values prior to analysis. Little’s MCAR test indicated that missing data were missing completely at random (χ^2^ = 143.27, df = 128, *p* = 0.17). Missing values were handled using full information maximum likelihood (FIML) estimation.

Preliminary analyses including descriptive statistics, internal consistency estimates, bivariate correlations, and independent samples t-tests comparing males and females were conducted using SPSS 27.0. The primary analysis employed structural equation modeling (SEM) with Mplus 8.8 using maximum likelihood estimation with robust standard errors (MLR). Model fit was evaluated using the following criteria: Comparative Fit Index (CFI) ≥ 0.90, Tucker-Lewis Index (TLI) ≥ 0.90, Root Mean Square Error of Approximation (RMSEA) ≤ 0.08, and Standardized Root Mean Square Residual (SRMR) ≤ 0.08 (Hu and Bentler, 1999).

For the chain mediation analysis, we specified paths from parental expectations to basic psychological need satisfaction, from parental expectations to autonomous motivation (RAI), from need satisfaction to autonomous motivation, and from parental expectations, need satisfaction, and autonomous motivation to exercise behavior (log-transformed MET-min/week). Because every construct in the structural portion of the model is connected by a directed path, this structural sub-model is just-identified; global model fit therefore reflects the measurement portion, and the structural estimates reproduce the observed associations among the four constructs. Indirect effects were tested using bias-corrected bootstrap confidence intervals with 5,000 resamples. Four specific indirect pathways were examined: (a) Parental Expectations → BP*N* → Exercise Behavior (Indirect 1); (b) Parental Expectations → BP*N* → RAI → Exercise Behavior (Indirect 2, the chain-mediated path); (c) Parental Expectations → RAI → Exercise Behavior (Indirect 3); and (d) the total indirect effect (Indirect 1 + Indirect 2 + Indirect 3). Estimates are reported in both unstandardized (b) and standardized (β) metrics.

For the gender moderation analysis, multi-group SEM was conducted comparing male and female subsamples. Measurement invariance was evaluated through a series of nested multi-group models: configural invariance (identical factor structure across groups), metric invariance (factor loadings constrained equal across groups), and scalar invariance (item intercepts additionally constrained equal, with the two latent means freed in the female group, yielding 16 net additional constraints). Because the RAI and exercise behavior were observed composite scores, invariance testing concerned the measurement parameters of the two latent constructs (parental expectations and basic psychological need satisfaction) and was conducted within the multi-group version of the structural model described above. Invariance was considered supported when the change in CFI did not exceed 0.01 and the change in RMSEA did not exceed 0.015 (Chen, 2007). Because MLR estimation was used, nested model comparisons employed Satorra-Bentler scaled chi-square difference tests computed from the MLR scaling correction factors (Satorra and Bentler, 2001); gender differences in specific structural paths were tested with the same scaled difference tests. The gender difference in the chain-mediated indirect effect was tested by directly estimating the between-group difference as a model constraint and computing a bias-corrected bootstrap confidence interval (5,000 resamples) for that difference; the difference was considered statistically significant when its confidence interval excluded zero.

A sensitivity analysis was conducted to evaluate the adequacy of the sample size. With *N* = 518, the study provides 80% power to detect bivariate correlations as small as *r* = 0.12 and standardized path coefficients of approximately 0.12 at α = 0.05 (two-tailed). In addition, a Monte Carlo simulation (2,000 replications) parameterized by the observed standardized path coefficients, with autonomous motivation regressed on both parental expectations and need satisfaction, indicated statistical power exceeding 0.99 for detecting the chain-mediated indirect effect of the observed magnitude, and 80% power for chain-mediated indirect effects as small as 0.025 in standardized units.

## 4 Results

### 4.1 Preliminary analyses

Table 1 presents the descriptive statistics, internal consistency coefficients, and bivariate correlations among the main study variables. All variables approximated normality (skewness < |1.2|, kurtosis < |1.8|). Perceived parental expectations were positively correlated with basic psychological need satisfaction (*r* = 0.35, *p* < 0.001), autonomous motivation (*r* = 0.27, *p* < 0.001), and exercise behavior (*r* = 0.24, *p* < 0.001). Basic psychological need satisfaction was positively correlated with autonomous motivation (*r* = 0.44, *p* < 0.001) and exercise behavior (*r* = 0.31, *p* < 0.001). Autonomous motivation was positively correlated with exercise behavior (*r* = 0.37, *p* < 0.001).

**Table 1:** Descriptive statistics, internal consistency, correlations, and gender comparisons (*N* = 518).

Variable	α	1	2	3	4	Male M (SD)	Female M (SD)	*t*	Cohen’s *d*
1. Parental expectations	0.86	—				3.52 (0.76)	3.72 (0.73)	−3.04**	−0.26
2. BPN satisfaction	0.90	0.35***	—			3.58 (0.69)	3.34 (0.74)	3.80***	0.35
3. Autonomous motivation (RAI)	0.86	0.27***	0.44***	—		3.45 (3.16)	4.16 (2.94)	−2.63*	−0.22
4. Exercise behavior (log MET)	—	0.24***	0.31***	0.37***	—	3.42 (0.33)	3.23 (0.35)	6.34***	0.58

BPN, basic psychological needs; RAI, Relative Autonomy Index; MET, metabolic equivalent of task. Correlations below diagonal (variables 1–4). t and Cohen’s *d* values represent male-minus-female contrasts; negative values indicate higher female means. *p < 0.05; **p < 0.01; ***p < 0.001.

Independent samples t-tests comparing male and female adolescents (see Table 1) revealed that females reported significantly higher perceived parental expectations for physical activity (*d* = −0.26, *p* = 0.003) and higher autonomous motivation (*d* = −0.22, *p* = 0.011). Conversely, males reported significantly higher levels of exercise behavior (*d* = 0.58, *p* < 0.001) and basic psychological need satisfaction in exercise (*d* = 0.35, *p* < 0.001). Cohen’s d was computed as the male mean minus the female mean divided by the pooled standard deviation, such that negative values indicate higher female means.

### 4.2 Measurement model

A confirmatory factor analysis (CFA) was conducted to evaluate the measurement model. Because the RAI is a weighted composite calculated from the five BREQ-2 regulation scores and exercise behavior is a single log-transformed MET-min/week score, these two variables were entered in the structural models as observed composite scores rather than latent factors. The measurement model therefore comprised two latent constructs: parental expectations (six items) and basic psychological need satisfaction (12 items), totaling 18 item indicators. The model was identified by fixing the first loading of each factor to 1.0 and freely estimating the factor variances; with 171 distinct sample moments and 37 free parameters (16 factor loadings, 18 residual variances, two factor variances, and one factor covariance), the model had 134 degrees of freedom. The two-factor model demonstrated acceptable fit: χ^2^(134) = 287.04, *p* < 0.001, CFI = 0.94, TLI = 0.93, RMSEA = 0.047 [90% CI: 0.039, 0.055], SRM*R* = 0.046. All standardized factor loadings were significant (*p* < 0.001) and exceeded 0.50, supporting convergent validity.

### 4.3 Chain mediation analysis

The hypothesized chain mediation model provided good fit to the data: χ^2^(166) = 355.95, *p* < 0.001, CFI = 0.944, TLI = 0.935, RMSEA = 0.047 [90% CI: 0.040, 0.054], SRM*R* = 0.049. Figure 1 displays the standardized path coefficients for the full sample model. Because all variables were assessed at a single time point, the path coefficients and indirect effects reported below represent model-based associations whose temporal ordering is derived from theory rather than demonstrated by the design.

Parental expectations positively predicted basic psychological need satisfaction (*β* = 0.35, *p* < 0.001, R^2^ = 0.12). Autonomous exercise motivation was predicted by both need satisfaction (*β* = 0.39, *p* < 0.001) and, to a smaller degree, parental expectations (*β* = 0.13, *p* = 0.002), the two antecedents jointly explaining 21% of its variance. Autonomous motivation, in turn, significantly predicted exercise behavior (*β* = 0.27, *p* < 0.001). Parental expectations retained a significant direct association with exercise behavior (*β* = 0.11, *p* = 0.008), and need satisfaction was directly associated with exercise behavior beyond the motivational pathway (*β* = 0.15, *p* = 0.001). The model explained 17% of the variance in exercise behavior.

Bootstrap mediation analysis (Table 2) revealed significant indirect effects. The total indirect effect of parental expectations on exercise behavior was *b* = 0.06, 95% CI [0.04, 0.08], *p* < 0.001 [*β* = 0.13, 95% CI (0.08, 0.17)]. The chain-mediated pathway (Parental Expectations → BP*N* → RAI → Exercise Behavior) was significant: *b* = 0.02, 95% CI [0.01, 0.03], *p* < 0.001 [*β* = 0.04, 95% CI (0.02, 0.05)]. The pathway through need satisfaction alone (Parental Expectations → BP*N* → Exercise Behavior, bypassing motivation) was also significant: *b* = 0.02, 95% CI [0.01, 0.04], *p* = 0.003 [*β* = 0.05, 95% CI (0.02, 0.09)], as was the pathway through autonomous motivation alone (Parental Expectations → RAI → Exercise Behavior): *b* = 0.02, 95% CI [0.01, 0.03], *p* = 0.005 [*β* = 0.04, 95% CI (0.01, 0.06)]. The three specific indirect effects sum to the total indirect effect (0.02 + 0.02 + 0.02 = 0.06 unstandardized; 0.05 + 0.04 + 0.04 = 0.13 standardized), which together with the direct effect (*b* = 0.05; *β* = 0.11) yields a total effect of *b* = 0.11 (*β* = 0.24). Because the structural sub-model is just-identified, this total effect exactly reproduces the zero-order correlation between parental expectations and exercise behavior (*r* = 0.24), providing an internal consistency check on the effect decomposition. Based on the unrounded estimates, the total indirect effect accounted for 52.6% of the total effect, and the chain-specific pathway accounted for 15.7% of the total effect. These results support Hypotheses 1 and 2, indicating that parental expectations are associated with adolescent exercise behavior both directly and indirectly through the chain mediation of basic psychological need satisfaction and autonomous motivation.

**Table 2:** Bootstrap analysis of indirect effects (5,000 Resamples): unstandardized (b) and standardized (β) estimates.

Indirect pathway	*b*	95% CI	*β*	95% CI	*p*
PE → BP*N* → Exercise (Indirect 1)	0.02	[0.01, 0.04]	0.05	[0.02, 0.09]	0.003
PE → BP*N* → RAI → Exercise (Indirect 2: Chain)	0.02	[0.01, 0.03]	0.04	[0.02, 0.05]	<0.001
PE → RAI → Exercise (Indirect 3)	0.02	[0.01, 0.03]	0.04	[0.01, 0.06]	0.005
Total indirect effect	0.06	[0.04, 0.08]	0.13	[0.08, 0.17]	<0.001
Direct effect (PE → Exercise)	0.05	[0.01, 0.09]	0.11	[0.03, 0.20]	0.008
Total effect (Direct + Indirect)	0.11	[0.07, 0.15]	0.24	[0.16, 0.32]	<0.001

PE, parental expectations; BPN, basic psychological need satisfaction; RAI, Relative Autonomy Index. b, unstandardized coefficient; β, standardized coefficient; bias-corrected bootstrap confidence intervals (5,000 resamples); standardized estimates and interval limits were rescaled via the ratio of standard deviations. The three specific indirect effects sum to the total indirect effect, and the total effect equals the total indirect effect plus the direct effect; the total effect equals the zero-order PE–exercise correlation (*r* = 0.24), as required by the just-identified structural sub-model. Proportions mediated were computed from unrounded estimates: total indirect effect / total effect = 52.6%; chain-specific indirect effect / total effect = 15.7%.

### 4.4 Gender moderation analysis

Multi-group SEM was conducted to test whether the chain-mediated pathway differed by gender. Measurement invariance of the two latent constructs was supported across the male and female groups (Table 3). The configural model, in which the full structural model was estimated separately in each group without equality constraints, had 332 degrees of freedom (twice the 166 degrees of freedom of the single-sample structural model, rather than twice the 134 of the measurement-only CFA, because the structural regressions among the four constructs were estimated simultaneously) and showed acceptable fit, χ^2^(332) = 533.87, CFI = 0.927, TLI = 0.917, RMSEA = 0.049, SRM*R* = 0.052. Constraining the 16 factor loadings to equality across groups (metric invariance) did not significantly worsen fit, χ^2^(348) = 559.40, Satorra-Bentler scaled Δχ^2^(16) = 21.84, *p* = 0.148 (scaling correction factors 1.082 vs. 1.086, test correction c*d* = 1.169; unscaled difference 25.53), ΔCFI = −0.004, ΔRMSEA = 0.000. Constraining the 18 item intercepts to equality while freeing the two latent means in the female group (scalar invariance; 16 net additional constraints) also yielded acceptable fit, χ^2^(364) = 589.93, scaled Δχ^2^(16) = 25.44, *p* = 0.062 (scaling correction factor 1.091, c*d* = 1.200; unscaled difference 30.53), ΔCFI = −0.005, ΔRMSEA = 0.000. All changes in fit indices fell within the recommended criteria (Chen, 2007), supporting full metric and scalar invariance and justifying the comparison of structural parameters across gender.

**Table 3:** Measurement invariance across gender: fit indices for nested multi-group models.

Model	χ^2^	df	CFI	TLI	RMSEA	SRMR	SB Δχ^2^ (Δdf)	ΔCFI
Configural	533.87	332	0.927	0.917	0.049	0.052	—	—
Metric	559.40	348	0.923	0.917	0.049	0.056	21.84 (16)	−0.004
Scalar	589.93	364	0.918	0.915	0.049	0.057	25.44 (16)	−0.005

Invariance testing concerned the two latent constructs (parental expectations, 6 items; basic psychological need satisfaction, 12 items); the RAI and exercise behavior were observed composite scores. Models were estimated within the multi-group structural model, so the configural model has 2 × 166 = 332 degrees of freedom. Metric invariance constrained 16 factor loadings to equality across groups; scalar invariance additionally constrained 18 item intercepts while freeing the two latent means in the female group (16 net constraints). Δχ^2^ values are Satorra-Bentler scaled chi-square difference statistics computed from the MLR scaling correction factors (configural 1.082, metric 1.086, scalar 1.091; test correction factors c*d* = 1.169 and 1.200; unscaled differences 25.53 and 30.53). ΔRMSEA was 0.000 in both steps. Invariance criteria: ΔCFI ≤ 0.01 and ΔRMSEA ≤ 0.015 (Chen, 2007; Satorra and Bentler, 2001).

Gender differences emerged across several structural paths (Table 4). The path from basic psychological need satisfaction to autonomous motivation was significantly stronger for males (*β* = 0.46, *p* < 0.001) than females (*β* = 0.33, *p* < 0.001), Δχ^2^(1) = 5.87, *p* = 0.015. The path from autonomous motivation to exercise behavior was also stronger for males (*β* = 0.33, *p* < 0.001) than females (*β* = 0.21, *p* < 0.01), Δχ^2^(1) = 4.12, *p* = 0.042.

**Table 4:** Standardized structural path coefficients and indirect effects by gender.

Path	Male β	Female *β*	Δχ^2^(1)	*p*
PE → BPN	0.40***	0.30***	—	—
PE → RAI	0.15*	0.11	—	—
BP*N* → RAI	0.46***	0.33***	5.87	0.015
PE → Exercise (direct)	0.12	0.10	0.84	0.360
BP*N* → Exercise	0.17**	0.13*	—	—
RAI → Exercise	0.33***	0.21**	4.12	0.042
PE → BP*N* → Exercise	0.07**	0.04*	—	—
PE → BP*N* → RAI → Exercise (chain)	0.06 [0.03, 0.09]	0.02 [0.01, 0.04]	diff = 0.04 [0.01, 0.07]	0.020
PE → RAI → Exercise	0.05*	0.02	—	—

PE, parental expectations; BPN, basic psychological need satisfaction; RAI, Relative Autonomy Index. *β* = standardized coefficient; 95% bias-corrected bootstrap confidence intervals (5,000 resamples) in brackets for the chain pathway and its gender difference, the latter tested by direct estimation as a model constraint. Δχ^2^(1) = Satorra-Bentler scaled chi-square difference test for cross-group equality of the indicated path. R^2^ for exercise behavior: males 0.24, females 0.11. *p < 0.05; **p < 0.01; ***p < 0.001.

Critically, the chain-mediated indirect effect was significantly larger for males [effect = 0.06, 95% CI (0.03, 0.09)] than females [effect = 0.02, 95% CI (0.01, 0.04)]. The between-group difference in the chain-mediated indirect effect, estimated directly as a model constraint, was 0.04 with a bias-corrected bootstrap 95% CI of [0.01, 0.07], which excludes zero and indicates a statistically significant gender difference in the chain-mediated pathway. This finding supports Hypothesis 3, indicating that the sequential motivational mechanism linking parental expectations to exercise behavior is more robust for male adolescents. The direct effect of parental expectations on exercise behavior did not differ significantly by gender [Δχ^2^(1) = 0.84, *p* = 0.36].

### 4.5 Supplementary analyses

To rule out the possibility that gender differences in the chain-mediated pathway were attributable to differential measurement properties, we conducted sensitivity analyses excluding outliers and using alternative scoring methods for the IPAQ-SF (continuous MET-minutes without log transformation). The pattern of results remained substantively unchanged. Controlling for age, residential area, and family composition likewise did not alter the significance or direction of the reported effects (Supplementary Table S1); these variables serve only as rough proxies for family socioeconomic status, which was not directly assessed, and residual socioeconomic confounding cannot be excluded. As a diagnostic for common method variance, Harman’s single-factor test was conducted on all 37 items: the unrotated first factor accounted for 28.4% of the total variance, below the conventional 40% threshold, suggesting that a single method factor is unlikely to account for the observed associations.

## 5 Discussion

The present study is among the first to integrate the Expectancy-Value Model of parental socialization with Self-Determination Theory to elucidate the psychological mechanisms through which parental expectations for physical activity are associated with adolescents’ exercise behavior. Three principal findings emerged: (a) parental expectations positively predicted exercise behavior both directly and indirectly through the chain mediation of basic psychological need satisfaction and autonomous exercise motivation; (b) the total indirect effect accounted for just over half (52.6%) of the total effect, with the chain-specific pathway accounting for 15.7%, highlighting the centrality of motivational mechanisms in the parental expectation–exercise behavior association; and (c) gender moderated the chain-mediated pathway, with the indirect effect being substantially larger for males than females. Consistent with the cross-sectional design, these findings represent concurrent associations that are compatible with the proposed theoretical sequence rather than evidence of a demonstrated causal chain.

### 5.1 The chain mediation: from parental expectations to exercise behavior

The finding that basic psychological need satisfaction and autonomous exercise motivation sequentially mediate the parental expectation–exercise behavior relationship extends SDT to the domain of parental socialization in a theoretically meaningful way. Prior SDT research has demonstrated that autonomy-supportive contexts—primarily in educational and coaching settings—facilitate need satisfaction, autonomous motivation, and adaptive outcomes (Vasconcellos et al., 2020; Standage et al., 2003). The present findings are consistent with the proposition that this motivational sequence also operates within the family context: when adolescents perceive that their parents hold positive expectations for their physical activity engagement, they are more likely to experience satisfaction of their basic psychological needs during exercise, which in turn fosters the internalization of exercise motivation and is associated with actual exercise behavior.

The significant direct path from parental expectations to exercise behavior (*β* = 0.11) suggests that expectations may also operate through additional mechanisms not captured in the present model, such as behavioral modeling, logistical facilitation, and social norms communicated through family routines (Edwardson and Gorely, 2010). Nonetheless, the substantial total indirect pathway (*β* = 0.13, accounting for 52.6% of the total effect) highlights that psychological internalization processes are a major conduit of the parental expectation–behavior association. In interpreting these findings, it is important to distinguish the content and intensity of parental expectations, which the present measure captured, from the interpersonal style through which those expectations are communicated (autonomy-supportive versus controlling), which was not assessed. The observed associations therefore reflect the perceived strength of parental expectations per se, and the need-supportive mechanisms discussed above should be understood as theoretically grounded interpretations rather than directly tested processes.

Basic psychological need satisfaction also maintained a direct association with exercise behavior (*β* = 0.15) independent of the motivational pathway. This suggests that beyond its role in fostering autonomous motivation, need satisfaction may directly energize behavior through mechanisms such as enhanced vitality, reduced amotivation, and positive affective experiences during exercise (Ryan and Deci, 2017).

### 5.2 Gender moderation of the motivational pathway

The gender moderation finding—that the chain-mediated indirect effect was three times larger for males than females—is a novel contribution to the literature on parental influences on adolescent physical activity. While prior research has documented mean-level gender differences in physical activity (Sallis et al., 2000; Biddle et al., 2019) and differential parental treatment (Fredricks and Eccles, 2005), the present study identifies a qualitative difference in the motivational processes themselves.

Two interconnected mechanisms may underlie this gender difference. First, the two key structural paths—from need satisfaction to autonomous motivation and from autonomous motivation to exercise behavior—were both significantly stronger for males. This suggests that, for male adolescents, the psychological translation of need satisfaction into autonomous drive and of autonomous drive into behavioral enactment is more efficient. This may reflect gender-role socialization: because exercise is normatively embedded within the male gender role, boys may have developed stronger cognitive-behavioral links between exercise-related motivation and actual exercise participation (Fredricks and Eccles, 2005).

Second, for female adolescents, factors beyond motivation—such as perceived barriers (e.g., lack of time, self-consciousness, safety concerns), competing academic and social demands, and body image concerns—may attenuate the motivation-behavior link. The lower R^2^ for exercise behavior in the female model (R^2^ = 0.11 vs. R^2^ = 0.24 in males) is consistent with this interpretation, suggesting that additional variables not captured in the current model are needed to fully explain female adolescents’ exercise behavior. It should be noted that these candidate mechanisms—including perceived barriers, body image concerns, and gender-role attitudes—were not directly assessed in the present study and are offered as theoretically informed interpretations requiring further empirical examination. Future research should investigate whether variables such as body image, peer norms, and gender-role attitudes moderate or mediate the parental expectation–exercise behavior relationship among female adolescents.

### 5.3 Practical implications

The implications discussed below are suggested by the observed cross-sectional associations and should be understood as hypotheses to be tested in future longitudinal and intervention research, rather than as demonstrated intervention effects. First, the strong chain-mediated pathway indicates that simply increasing parental expectations is insufficient; parents must communicate their expectations in ways that support—rather than thwart—adolescents’ basic psychological needs. Parent education programs should equip parents with need-supportive communication strategies, including acknowledging adolescents’ perspectives on physical activity, offering meaningful choices in activity types, and providing competence-affirming feedback rather than evaluative pressure.

Second, the gender moderation findings suggest that one-size-fits-all approaches to parental influence are unlikely to be effective. For male adolescents, interventions that strengthen the parental expectation–need satisfaction–motivation pathway may yield behavioral benefits. For female adolescents, however, supplementary strategies—such as addressing perceived barriers, promoting positive body image, and leveraging peer support—may be necessary to complement parental expectations and bridge the motivation-behavior gap.

Third, in the Chinese educational context where academic demands are intense, helping parents recognize that physical activity expectations can coexist with and even support academic goals—given the well-documented cognitive and academic benefits of regular exercise (Lubans et al., 2016)—may be an effective messaging strategy for increasing parental buy-in.

### 5.4 Limitations and future directions

Several limitations of this study warrant consideration. First, the cross-sectional design precludes causal inference. While the proposed temporal ordering of the chain mediation model is theoretically grounded, reverse and reciprocal relationships are plausible. For example, adolescents who are more physically active may elicit higher parental expectations, creating a bidirectional feedback loop. Longitudinal designs with at least three waves of data collection are needed to establish temporal precedence and test reciprocal effects over time. Relatedly, the indirect effects and the proportion mediated reported here are model-based estimates whose interpretation depends on the assumed causal ordering; because the structural sub-model is just-identified, statistically equivalent models that reorder the two mediators or reverse individual structural paths reproduce identical fit statistics, so alternative orderings are statistically indistinguishable in cross-sectional data and await longitudinal testing.

Second, all variables were assessed via adolescent self-report during a single assessment occasion, introducing the possibility of common method variance that may inflate the observed associations. The findings concerning exercise behavior should therefore be understood specifically in terms of self-reported exercise behavior rather than objectively measured physical activity. Future studies should incorporate multi-informant assessments (e.g., parent-reported expectations, objectively measured physical activity via accelerometry) to strengthen methodological rigor. Third, the sample was drawn from a single city in central China, limiting generalizability to other regions and to rural populations where parental expectations and physical activity opportunities may differ substantially. Moreover, unmeasured confounders, such as family socioeconomic status, may have influenced several study variables simultaneously; although age, residential area, and family composition were statistically controlled, residual confounding cannot be excluded.

Fourth, the present study did not differentiate between autonomous and controlling forms of parental expectations. It is plausible that the need-thwarting effects of controlling parental expectations may be particularly detrimental to female adolescents, who may be more sensitive to evaluative pressure in the physical domain. Future research employing multidimensional assessments of parental expectations—distinguishing between supportive and pressuring forms—would provide a more nuanced understanding. In addition, the three basic psychological needs were combined into a single global need satisfaction score. Although this approach is consistent with prior SDT research and was supported by the intercorrelations among the subscales in the present sample, combining autonomy, competence, and relatedness may obscure need-specific processes, and future research should examine the three needs as distinct mediators.

Finally, the study focused exclusively on exercise behavior quantity (MET-min/week) and did not examine behavioral quality dimensions such as exercise adherence, enjoyment, or type of activity. Future research should investigate whether the chain mediation model extends to these important behavioral outcomes and whether gender moderates qualitatively different aspects of exercise engagement.

## 6 Conclusion

This study provides novel evidence that basic psychological need satisfaction and autonomous exercise motivation operate as sequential mediating mechanisms in the association between parental expectations and adolescents’ exercise behavior. The findings reveal that the indirect motivational pathways jointly account for just over half (52.6%) of the total association between parental expectations and exercise behavior, with the chain-specific pathway accounting for approximately one sixth (15.7%), and that these pathways operate differently for male and female adolescents. For male adolescents, the motivational pathway linking parental expectations to behavior is robust; for female adolescents, this pathway is attenuated, suggesting the need for gender-tailored intervention strategies. By integrating the Expectancy-Value Model of parental socialization with Self-Determination Theory, this research advances an understanding of the mechanisms through which parental expectations are associated with health behavior, and for whom this internalization process is most consequential.

## Data Availability

The raw data supporting the conclusions of this article will be made available by the authors, without undue reservation.
