# Supplementary material for: Parental expectations for physical activity and adolescents’ exercise behavior: a chain mediation of basic psychological needs and exercise motivation moderated by gender
Source: Front Psychol. 2026 Oct 19;17:1964799. doi: 10.3389/fpsyg.2026.1964799 (PMC13724339; doi:10.3389/fpsyg.2026.1964799)
Supplement: Supplementary file 1 [file Supplementary_file_1.docx]

**Supplementary Material**

Parental Expectations for Physical Activity and Adolescents’ Exercise Behavior: A Chain Mediation of Basic Psychological Needs and Exercise Motivation Moderated by Gender

*Shikai Feng — Frontiers in Psychology, Sport Psychology*

**Supplementary Table S1. Covariate-Adjusted Models**

Standardized path coefficients and indirect effects before and after statistically controlling for age, residential area (urban vs. suburban), and family composition (two-parent, single-parent, or other household type). 95% bias-corrected bootstrap confidence intervals (5,000 resamples) in brackets.

| **Effect** | **Unadjusted β** | **Adjusted β** |
| --- | --- | --- |
| PE → BPN (a1) | .35*** | .34*** |
| PE → RAI (a2) | .13** | .12* |
| BPN → RAI (d21) | .39*** | .38*** |
| PE → Exercise, direct (c′) | .11** | .10* |
| BPN → Exercise (b1) | .15*** | .14** |
| RAI → Exercise (b2) | .27*** | .26*** |
| Indirect: PE → BPN → Exercise | .05 [.02, .09] | .05 [.02, .08] |
| Indirect: PE → BPN → RAI → Exercise (chain) | .04 [.02, .05] | .03 [.02, .05] |
| Indirect: PE → RAI → Exercise | .04 [.01, .06] | .03 [.01, .06] |
| Total indirect effect | .13 [.08, .17] | .11 [.07, .16] |
| Total effect | .24 [.16, .32] | .21 [.13, .29] |

*Note. PE = parental expectations; BPN = basic psychological need satisfaction; RAI = Relative Autonomy Index. Adjusted models include age, residential area, and family composition as covariates predicting BPN, RAI, and exercise behavior. Residential area and family composition serve only as rough proxies for family socioeconomic status. *p < .05; **p < .01; ***p < .001.*

**S2. Mplus 8.8 Input Syntax**

S2.1 Full-sample chain mediation model (Tables 2 and 3, Figure 1)

```
TITLE: BF002 chain mediation, full sample (N = 518);
DATA:  FILE IS bf002_full.dat;
VARIABLE:
  NAMES ARE id gender pe1-pe6 bpn1-bpn12 rai logmet age urban famcomp;
  USEVARIABLES ARE pe1-pe6 bpn1-bpn12 rai logmet;
  MISSING ARE ALL (-999);
ANALYSIS:
  ESTIMATOR = MLR;
  BOOTSTRAP = 5000;
MODEL:
  PE  BY pe1-pe6;
  BPN BY bpn1-bpn12;
  BPN ON PE (a1);
  RAI ON PE (a2)
         BPN (d21);
  logmet ON PE (cp)
            BPN (b1)
            RAI (b2);
MODEL CONSTRAINT:
  NEW(ind_bpn ind_rai ind_chain ind_total direct total p_ind p_chain);
  ind_bpn   = a1*b1;
  ind_rai   = a2*b2;
  ind_chain = a1*d21*b2;
  ind_total = a1*b1 + a2*b2 + a1*d21*b2;
  direct    = cp;
  total     = cp + ind_total;
  p_ind     = ind_total/total;
  p_chain   = ind_chain/total;
OUTPUT: STDYX TECH1 CINTERVAL(BCBOOTSTRAP);
```

S2.2 Multi-group model and measurement invariance (Tables 3 and 4)

```
TITLE: BF002 multi-group model, configural/metric/scalar invariance;
DATA:  FILE IS bf002_full.dat;
VARIABLE:
  NAMES ARE id gender pe1-pe6 bpn1-bpn12 rai logmet age urban famcomp;
  USEVARIABLES ARE pe1-pe6 bpn1-bpn12 rai logmet;
  GROUPING IS gender (1 = male  2 = female);
  MISSING ARE ALL (-999);
ANALYSIS:
  ESTIMATOR = MLR;
  MODEL = CONFIGURAL METRIC SCALAR;
  BOOTSTRAP = 5000;
MODEL:
  PE  BY pe1-pe6;
  BPN BY bpn1-bpn12;
  BPN ON PE (a1);
  RAI ON PE (a2)
         BPN (d21);
  logmet ON PE (cp)
            BPN (b1)
            RAI (b2);
MODEL female:
  BPN ON PE (a1f);
  RAI ON PE (a2f)
         BPN (d21f);
  logmet ON PE (cpf)
            BPN (b1f)
            RAI (b2f);
MODEL CONSTRAINT:
  NEW(chain_m chain_f chain_diff);
  chain_m    = a1*d21*b2;
  chain_f    = a1f*d21f*b2f;
  chain_diff = chain_m - chain_f;
OUTPUT: STDYX TECH1 CINTERVAL(BCBOOTSTRAP);

! Note. Scalar invariance constrains the 18 item intercepts equal across
! groups and frees the two latent means (PE, BPN) in the female group,
! yielding 16 net additional constraints. Chi-square difference tests use
! the Satorra-Bentler scaled difference statistic (MLR scaling correction
! factors: configural 1.082, metric 1.086, scalar 1.091; cd = 1.169, 1.200).
! Covariate-adjusted models (Table S1) add "ON age urban famcomp" to the
! BPN, RAI, and logmet regressions.
```

**S3. Monte Carlo Power Simulation Code**

Python 3.12 code used for the power sensitivity analysis reported in Section 3.3 (2,000 replications, N = 518 per replication, parameters fixed at the observed standardized solution).

```
import math
import numpy as np
from scipy import stats as st

N, REPS = 518, 2000
a1, a2, d21 = .350, .132, .394      # X->M1, X->M2, M1->M2
cp, b1, b2  = .114, .150, .273      # X->Y,  M1->Y, M2->Y
R2_M1, R2_M2, R2_Y = .122, .209, .175

rng = np.random.default_rng(20261004)
hits, ests = 0, []
se_a1  = math.sqrt((1 - R2_M1) / (N - 2))
se_d21 = math.sqrt((1 - R2_M2) / (N - 3))
se_b2  = math.sqrt((1 - R2_Y)  / (N - 4))

for _ in range(REPS):
    X  = rng.normal(size=N)
    M1 = a1*X + rng.normal(scale=math.sqrt(1 - R2_M1), size=N)
    M2 = a2*X + d21*M1 + rng.normal(scale=math.sqrt(1 - R2_M2), size=N)
    Y  = cp*X + b1*M1 + b2*M2 + rng.normal(scale=math.sqrt(1 - R2_Y), size=N)
    Xs  = (X - X.mean()) / X.std()
    M1s = (M1 - M1.mean()) / M1.std()
    M2s = (M2 - M2.mean()) / M2.std()
    Ys  = (Y - Y.mean()) / Y.std()
    h_a1  = np.polyfit(Xs, M1s, 1)[0]
    h_d21 = np.linalg.lstsq(np.c_[Xs, M1s, np.ones(N)], M2s, rcond=None)[0][1]
    h_b2  = np.linalg.lstsq(np.c_[Xs, M1s, M2s, np.ones(N)], Ys, rcond=None)[0][2]
    chain = h_a1 * h_d21 * h_b2
    ests.append(chain)
    se = math.sqrt((h_d21*h_b2*se_a1)**2 + (h_a1*h_b2*se_d21)**2
                   + (h_a1*h_d21*se_b2)**2)
    if abs(chain / se) > 1.96:
        hits += 1

ests = np.array(ests)
power = hits / REPS                       # = 1.000
min_chain = (st.norm.ppf(.975) + st.norm.ppf(.80)) * ests.std()   # = .024
print(f"power = {power:.3f}; 80%-detectable chain = {min_chain:.3f}")
```
